# Phytobiotics with Adsorbent to Mitigate Toxicity of Multiple Mycotoxins on Health and Growth of Pigs

**DOI:** 10.3390/toxins13070442

**Published:** 2021-06-26

**Authors:** Debora Muratori Holanda, Young Ihn Kim, Wanpuech Parnsen, Sung Woo Kim

**Affiliations:** Department of Animal Science, North Carolina State University, Raleigh, NC 27695, USA; dmurato@ncsu.edu (D.M.H.); ykim28@ncsu.edu (Y.I.K.); wparnse@ncsu.edu (W.P.)

**Keywords:** aflatoxin, deoxynivalenol, fumonisin, growth, health, phytobiotic, pig

## Abstract

Phytobiotics with a mycotoxin adsorbent were used to mitigate negative effects of multiple mycotoxins in diets fed to pigs. In experiment 1, 120 pigs (11.6 kg body weight; BW) were assigned to five treatments (three pigs/pen) and fed for 28 days. Treatments were CON (control), MTD (CON + 2.5 mg/kg of deoxynivalenol), DP (MTD + phytobiotics at 0.1%), and DPA1 and DPA2 (MTD + phytobiotics and adsorbent at 0.1% and 0.2%, respectively). In experiment 2, 96 pigs (28.5 kg BW) were assigned to four treatments (three pigs/pen) and fed for 26 days. Treatments were CON, MTAF (CON + 0.19 mg/kg of aflatoxin and 8 mg/kg of fumonisins), AFP (MTAF + phytobiotics at 0.1%), and AFPA (MTAF + phytobiotics and adsorbent at 0.1%). Growth performance was measured weekly, and blood was sampled at the end of study to measure hepatic function and inflammatory status (TNF-α). Data were analyzed using the MIXED procedure. In experiment 1, pigs fed MTD, DP, DPA1, and DPA2 had smaller (*p* < 0.05) BW than CON. Pigs fed DPA2 had greater (*p* < 0.05) BW than MTD. Pigs fed DP and DPA2 tended to have lower (*p* < 0.1) serum total protein than CON. Pigs fed MTD and DPA2 tended to have higher (*p* < 0.1) alanine aminotransferase than CON. Similarly, pigs fed MTD, DP, and DPA2 tended to have higher (*p* < 0.1) urea nitrogen/creatinine than CON. In experiment 2, pigs fed MTAF, AFP, and AFPA had smaller (*p* < 0.05) BW than CON. Pigs fed MTAF, AFP, and AFPA had smaller (*p* < 0.05) ADFI than CON. Pigs fed AFPA had higher (*p* < 0.05) aspartate aminotransferase than CON and MTAF. Pigs fed AFP and AFPA had higher (*p* < 0.05) alanine aminotransferase than CON. Pigs fed MTAF, AFP, and AFPA had lower (*p* < 0.05) urea nitrogen/creatinine than CON. Pigs fed AFPA had higher (*p* < 0.05) TNF-α than CON and MTAF. In conclusion, feeding an additional 2.5 mg/kg of deoxynivalenol or 0.19 mg/kg of aflatoxin with 8 mg/kg of fumonisins reduced the growth of pigs. Deoxynivalenol compromised the hepatic function of pigs. Phytobiotics with adsorbent could partly overcome the detrimental effects of mycotoxins.

## 1. Introduction

Mycotoxins are metabolites from fungi commonly found as contaminants in feedstuffs and feeds globally [1]. Among mycotoxins, aflatoxins, fumonisins, and deoxynivalenol pose a higher risk for pigs due to their higher occurrence, detrimental effects, and species susceptibility [2,3]. The detrimental effects of aflatoxins, deoxynivalenol, and fumonisins in pigs include immune modulation [4,5], cell toxicity [4], increase in oxidative stress and inflammatory markers [6,7], and reduction in feed intake with eventual decrease in growth [8,9]. In order to reduce damages from these mycotoxins, their levels in feeds are controlled by governmental regulation. In the United States, limitations are 10 mg/kg of feeds for fumonisins (sum of B1, B2, and B3) [10] and 0.02 and 0.20 mg/kg for aflatoxins for nursery and growing pigs, respectively [11], whereas the advisory level for deoxynivalenol is 1 mg/kg [12]. Similarly, the European Union specifies limits for fumonisins (sum of B1 and B2) at 5 mg/kg [13] and for aflatoxin B1 at 0.01 and 0.02 mg/kg for nursery and older pigs, respectively [14], whereas the advisory level for deoxynivalenol is 0.9 mg/kg [13].

From recent surveys and reviews, more than 70% of feedstuffs are shown to contain multiple mycotoxins at levels affecting the health of animals [1,2,3]. In order to handle issues with prevalent mycotoxins, feed additives with the ability to counteract the toxic effects have been investigated and used in pig production. These feed additives mostly include multiple bioactive components as absorbent [15,16], gut immune modulators [4,8,17], and detoxifiers [18,19]. Recently, bioactive components with antioxidative and anti-inflammatory properties have been shown to be effective by reducing gut distress from mycotoxins in porcine in vitro models [20,21], suggesting the need for in vivo validation.

Phytobiotics are well reviewed for their antioxidative and anti-inflammatory properties [22,23,24]. Phytobiotics typically include bioactive extracts from plants, algae, and their derivatives, such as essential oils and aromatic herbs [23]. The importance of phytobiotics for the livestock industry rose with the ban of antibiotics as growth promoters and the increased interest in organic production of animals. Overall, phytobiotics have shown the ability to improve growth performance by increasing feed intake and to enhance health by reducing inflammation and oxidative stress, as well as microbial infection, especially related to the function of phenolic compounds in phytobiotics [25,26]. Thus, the properties of phytobiotics could enable their use in mycotoxin-contaminated feeds to reduce the detrimental effects of mycotoxins to pigs.

The aim of the current study was to evaluate the potential mitigation effects by phytobiotics and mycotoxin adsorbent in feed naturally contaminated with mycotoxins on the growth and health of nursery and growing pigs.

## 2. Results

### 2.1. Growth Performance

In experiment 1, the initial body weight of pigs was not different (*p* > 0.05) among dietary treatments (Table 1). On day 7, the lack of a difference (*p* > 0.05) in the body weight of pigs among treatments was maintained. Pigs fed MTD, DP, DPA1, and DPA2 showed reduced (*p* < 0.05) body weight compared with pigs fed CON on day 14. On day 21, pigs fed MTD, DP, DPA1, and DPA2 showed reduced (*p* < 0.05) body weight compared with pigs fed CON. However, pigs fed DPA1 and DPA2 showed increased (*p* < 0.05) body weight compared to MTD. On day 28, pigs fed MTD, DP, DPA1, and DPA2 showed reduced (*p* < 0.05) body weight compared with pigs fed CON. However, pigs fed DPA2 showed increased (*p* < 0.05) body weight compared to MTD. There were no differences (*p* > 0.05) in the average daily gain (ADG) of pigs among treatments on days 0 to 7. Pigs fed MTD, DP, DPA1, and DPA2 had lower ADG (*p* < 0.05) than pigs fed CON on days 7 to 14, days 14 to 21, days 21 to 28, and in the overall period. On days 14 to 21 and in the overall period, pigs fed DPA2 had higher ADG (*p* < 0.05) than pigs fed MTD. On days 21 to 28, pigs fed DPA1 had lower ADG (*p* < 0.05) than pigs fed MTD and DPA2. For average daily feed intake (ADFI), the tendency for lower (*p* = 0.055) ADFI observed in pigs fed MTD and DP was recovered in pigs fed DPA1 and DPA2 from days 0 to 7. There were no differences in the ADFI of pigs among treatments in the other periods. Pigs fed MTD, DP, DPA1, and DPA2 had a lower (*p* < 0.05) gain–feed ratio than CON on days 7 to 14. At the same time, pigs fed DP had a lower (*p* < 0.05) gain–feed ratio than MTD. In the overall period, pigs fed MTD, DP, DPA1, and DPA2 had a lower (*p* < 0.05) gain–feed ratio than CON.

In experiment 2, no difference (*p* > 0.05) was observed in the body weight of pigs on day 0 (Table 2). On day 7, the lack of a difference (*p* > 0.05) in the body weight of pigs among treatments was maintained. Pigs fed MTAF, AFP, and AFPA had reduced (*p* < 0.05) body weight compared to CON on days 14, 21, and 26. There were no differences (*p* > 0.05) in the ADG of pigs among treatments on days 0 to 7, days 14 to 21, and days 21 to 26. Pigs fed MTAF, AFP, and AFPA had reduced (*p* < 0.05) ADG compared to CON on days 7 to 14 and in the overall period. Pigs fed AFPA tended to have lower (*p* = 0.061) ADFI than CON on days 0 to 7. Pigs fed MTAF, AFP, and AFPA had reduced (*p* < 0.05) ADFI than CON on days 7 to 14, days 14 to 21, days 21 to 26, and in the overall period. There were no differences (*p* > 0.05) in the gain–feed ratio of pigs among treatments during all periods assessed.

### 2.2. Blood Analyses

In experiment 1, pigs fed DP and DPA2 tended to show lower (*p* < 0.1) total protein in serum than CON (Table 3). Pigs fed MTD and DPA2 tended to show higher (*p* < 0.1) alanine aminotransferase than CON. Furthermore, pigs fed DPA2 tended to show higher (*p* < 0.1) alanine aminotransferase than DP. Pigs fed MTD, DP, DPA1, and DPA2 tended to show higher (*p* < 0.1) urea N than CON. Similarly, pigs fed MTD, DP, and DPA2 tended to show higher (*p* < 0.1) urea N/creatinine than CON. Pigs fed MTD, DP, DPA1, and DPA2 tended to show lower (*p* < 0.1) phosphorus than CON. Lastly, pigs fed MTD tended to show lower (*p* < 0.1) phosphorus than DP, DPA1, and DPA2. There were no differences in other blood variables of pigs among treatments.

In experiment 2, pigs fed AFPA had increased (*p* < 0.05) aspartate aminotransferase compared to CON and MTAF (Table 4). Pigs fed AFP and AFPA had increased (*p* < 0.05) alanine aminotransferase compared to CON. Pigs fed MTAF, AFP, and AFPA had higher (*p* < 0.05) creatinine but lower (*p* < 0.05) urea N/creatinine than CON. Pigs fed MTAF and AFPA had lower (*p* < 0.05) cholesterol than CON, whereas AFPA had lower (*p* < 0.05) cholesterol than AFP. Regarding immune variables, the only difference observed was in pigs fed AFPA, which had increased (*p* < 0.05) tumor necrosis factor-α compared to CON and MTAF. There were no differences (*p* > 0.05) in other blood variables of pigs among treatments.

## 3. Discussion

The phytobiotics (Vilocym) and a blend of phytobiotics with adsorbents (Vilocym Z) tested as feed additives in the current study were previously investigated in broilers [27] and nursery pigs [28] when animals were not challenged with mycotoxins, showing their ability to improve intestinal health. The same compounds were then tested in broilers [29] and laying hens [30] challenged with aflatoxins and ochratoxins, showing beneficial outcomes and the ability to overcome mycotoxin detrimental effects on animal performance. Therefore, further investigations regarding the ability of the aforementioned feed additives to overcome mycotoxin challenge in pigs are of interest. To the best of our knowledge, no study investigating the use of similar phytobiotics in pig diets challenged with mycotoxins has been published to date, and the current experiments show the importance of these phytobiotics in both nursery and growing pigs.

Mycotoxins are known to cause detrimental effects in pigs. Such effects are commonly observed when mycotoxin concentrations are above a certain threshold. Therefore, many countries have limited concentrations or have set advisory levels to hinder the toxic effects of mycotoxins. For deoxynivalenol, the United States has set advisory levels of lower than 1 mg/kg of diet, whereas, in Europe, it is advised that deoxynivalenol concentration should not exceed 0.9 mg/kg. In the United States, the upper limit for aflatoxins is 0.02 and 0.2 mg/kg for nursery and growing animals, respectively [11], whereas the upper limit for aflatoxin B1 in Europe is 0.01 and 0.02 mg/kg for nursery and older pigs, respectively [14]. Regarding fumonisins, the upper limit is 10 mg/kg in the United States [10] and 5 mg/kgs in Europe [13]. In experiment 1, pigs were successfully challenged with deoxynivalenol where the concentrations in CON (0.3 mg/kg) and the average for mycotoxin contaminated diets (2.7 mg/kg) were below and above the advisory levels, respectively. Similarly, in experiment 2, both aflatoxin B1 and fumonisin concentrations in CON (0 and 0 mg/kg, respectively) and the average for mycotoxin contaminated diets (0.26 and 8 mg/kg, respectively) were below or above the preconized limits by governmental authorities, respectively (for fumonisins, only above the levels set in Europe).

In experiment 1, the detrimental effects of deoxynivalenol (additional 2.5 mg/kg of feed) in pigs could be seen from the second week on ADG and from day 14 on BW, decreasing both variables in comparison to CON. Similar results were previously observed in nursery pigs challenged with 2 mg/kg of deoxynivalenol, where reduced ADG could be observed even sooner (days 0 to 7) [8]. As there were no differences in ADFI, the decrease in ADG and, eventually, BW was likely caused by a mechanism that decreased nutrient utilization for growth, as seen by the decreased feed efficiency (gain–feed ratio) in the overall period. Energy and nutrient digestibility were not assessed in the current study, but previous results have shown that pigs challenged with deoxynivalenol had lower apparent ileal digestibility for gross energy, dry matter, nitrogen, and ether extract [8], suggesting that an impaired digestion and absorption of feed components could justify the reduced growth observed in challenged pigs from experiment 1. Even though none of the feed additives tested could fully overcome deoxynivalenol effects on growth, the supplementation of diets contaminated with deoxynivalenol with phytobiotics and adsorbent at 0.2% improved pig ADG (days 14 to 21 and overall period) and BW (at the end of the study). It is possible that the blends of phytobiotics present in the feed additive could improve nutrient utilization by enhancing intestinal health of pigs, as seen in a previous study with broilers, where Vilocym Z increased villus height, villus height/crypt depth, and number of goblet cells in the jejunum [28]. This shows that pigs facing deoxynivalenol challenge in the current study (additional 2.5 mg/kg of feed) needed a combination of both phytobiotics and adsorbent and the higher concentration tested (0.2%) to hinder deoxynivalenol toxic effects.

In experiment 2, the same feed additives were tested in growing pigs challenged with aflatoxins and fumonisins. The detrimental effects of these mycotoxins were expected to be mitigated by the feed additives as pig susceptibility to mycotoxins decreases with age [4] and as the adsorbent in Vilocym Z, zeolite, has an adsorbing capacity of about 60% for aflatoxins [31,32] and about a 10-fold higher capacity to adsorb fumonisins than deoxynivalenol [33]. However, the detrimental effects of feeding aflatoxins and fumonisins (0.19 and 8 mg/kg mg/kg of feed, respectively) could be seen during the second week and overall period by a decrease in ADG regardless of the inclusion of phytobiotics and adsorbent. Such a decrease in ADG (−7.0%, −10.3%, and −10.0% for MTAF, AFP, and AFPA in comparison to CON, respectively) could be related to the similar decrease in ADFI (−9.9%, −10.2%, and −12.5% for MTAF, AFP, and AFPA in comparison to CON, respectively) in the overall period as no difference was observed for gain–feed ratio. As a result, pigs fed aflatoxins and fumonisins had a lower BW in comparison to CON. In contrast, another study showed that growing-finishing pigs fed aflatoxins and fumonisins (0.18 and 14 mg/kg, respectively) had a reduction in ADG and G:F, but without impacting their BW [4].

Regarding blood analyses, pigs in experiment 1 showed an increase in urea N when fed deoxynivalenol-contaminated diets in comparison to CON. Moreover, there was a tendency for decreased total protein in DP and DPA2 in comparison to CON and for increased alanine aminotransferase in MTD in comparison to CON. Such outcomes may suggest that protein synthesis was reduced and, thus, protein mobilization from body reserves was enhanced, with an increase in alanine aminotransferase, as a key enzyme in the Cori cycle, eventually increasing blood urea N [34]. Furthermore, deoxynivalenol has been related to impaired protein synthesis and hepatic damage [35], explaining the results observed for reduced total protein and increased alanine aminotransferase in blood [36]. The difference observed for urea N was reflected in urea N/creatinine, which showed a correspondent increase. Among minerals, the blood levels of phosphorus were the only ones influenced, showing a decrease in concentration in pigs fed deoxynivalenol. There are few scientific data published regarding the interaction between deoxynivalenol and calcium-to-phosphorus ratio, as well as its impact on vitamin D metabolism [8,37]; however, it seems that deoxynivalenol may affect the levels of such minerals via a mechanism still to be further investigated. The addition of phytobiotics or adsorbent to diets did not show ameliorating effects on blood variables of pigs challenged with deoxynivalenol.

In experiment 2, similar to the outcomes of experiment 1, pigs fed AFP and AFPA had higher alanine aminotransferase concentration in blood compared to CON. In addition, pigs fed AFPA had higher aspartate aminotransferase. The increase in hepatic enzymes indicates liver damage, which was previously reported after aflatoxin ingestion [15], suggesting damage to hepatocytes and release of intracellular enzymes. The liver damage is supported by the lower cholesterol concentration found in pigs fed MTAF and AFPA, as the liver is the main site for cholesterol synthesis [8,38]. On the other hand, unlike the result observed in experiment 1, there were no differences in urea N, but instead an increase in creatinine, resulting in a lower urea N/creatinine ratio. The increase in creatine may be suggestive of kidney failure [39]. Pigs challenged with fumonisins have shown impaired kidney function [40], and a lower urea N/creatinine ratio was observed in pigs challenged with aflatoxins [5]. A higher tumor necrosis factor-α concentration was observed in pigs fed AFPA in comparison to CON, suggesting systemic inflammation [41]. An increase in tumor necrosis factor-α was also observed in a previous study where pigs were challenged with aflatoxins and deoxynivalenol [6]. Unexpectedly, pigs fed AFPA also showed higher tumor necrosis factor-α concentration in comparison to MTAF. Such an outcome may indicate that the adsorbent had an effect of increasing systemic inflammation via a mechanism still to be uncovered.

## 4. Conclusions

Dietary mycotoxin challenge (2.5 mg/kg of deoxynivalenol in experiment 1 and 0.19 mg/kg of aflatoxin and 8 mg/kg of fumonisin in experiment 2) impaired the growth performance of pigs by reducing body weight gain and feed intake, disturbed liver and kidney function, and caused immune responses. The supplemental effects of phytobiotics (Vilocym) and a blend of phytobiotics with adsorbents (Vilocym Z) seem to be dependent on the types of mycotoxin and the dose level. This study shows that Vilocym Z at 0.2% enhanced the growth performance of pigs with deoxynivalenol challenge. However, Vilocym and Vilocym Z showed the potential to enhance immune response in pigs with mycotoxin challenges. This warrants further research to understand if the supplemental effects of Vilocym and Vilocym Z increasing the inflammatory response and hepatic enzyme are directly related to the gut health and gut microflora of pigs.

## 5. Materials and Methods

A protocol of this experiment was reviewed and approved by the Institutional Animal Care and Use Committee at North Carolina State University (Raleigh, NC, USA). The experiment was conducted at the North Carolina State University Swine Evaluation Station (Clayton, NC, USA).

### 5.1. Animals and Experimental Diets

In experiment 1, a total of 120 pigs (average body weight: 11.6 + 0.1 kg) were used in this experiment designed as a completely randomized block design. Pigs were allotted to five dietary treatments on the basis of sex (two sex blocks) and initial body weight (four body weight blocks). There were 10 pens/treatment and three pigs/pen. The dietary treatments were as follows: CON, control diet; MTD, CON + 2.5 mg/kg of deoxynivalenol; DP, MTD + phytobiotic additive (at 0.1%); DPA1, MTD + phytobiotic and adsorbent additive (at 0.1%); DPA2, MTD + phytobiotic and adsorbent additive (at 0.2%).

The phytobiotic additive (Vilocym, Ayurvet Limited, Ghaziabad, UP, India) was composed of plant derivatives (*Phyllanthus emblica*, *Curcuma longa*, *Allium sativum*, *Azadirachta indica*, *Withania somnifera*, *Trigonella foenumgraecum*, *Tinospora cordifolia*, *Andrographis paniculata*, *Phyllanthus niruri*, and *Boerhavia diffusa*). The phytobiotic and adsorbent additive (Vilocym Z, Ayurvet Limited, Ghaziabad, UP, India) was composed of the same blend of plant derivatives with additional acetic acid, propionic acid, and zeolite. The same batch of phytobiotic additive was used for both experiment 1 and experiment 2 in order to assure consistent dietary effects on pigs. The supplementation levels of phytobiotics were based on expected daily intake following previous studies with pigs [29] and laying hens [30].

Each dietary treatment had eight pen-replicates (four pens with barrows and four pens with gilts) with three pigs per pen. Pigs were fed the dietary treatment diets for 28 days. Distiller’s dried grains with solubles (DDGS) contaminated with deoxynivalenol (4.9 mg/kg; Table 5) were mixed with uncontaminated DDGS to achieve the targeted level of deoxynivalenol (2.5 mg/kg) in the diets MTD, DP, DPA1, and DPA2. Only uncontaminated DDGS was used in the CON. All diets were formulated to be isonitrogenous and isocaloric and meet the nutrient requirements for late nursery pigs (11 to 25 kg) as suggested by the National Research Council [42] (Table 6). Body weight and feed intake were measured weekly to obtain average daily gain, average daily feed intake, and gain–feed ratio.

Vilocym or Vilocym-Z was supplemented at 0.1% to the diets replacing the equal amount of corn.

In experiment 2, a total of 96 pigs were used in this experiment designed as a completely randomized block design. Pigs (average body weight: 28.5 + 0.2 kg) were randomly allotted to four dietary treatments on the basis of sex (two sex blocks) and initial body weight (four initial body weight blocks). Each dietary treatment had eight pen-replicates (four pens with barrows and four pens with gilts) with three pigs per pen. The dietary treatments were as follows: CON, control diet; MTAF, CON + 0.19 mg/kg of aflatoxin (B1 + B2) and 8.0 mg/kg of fumonisin B1; AFP, MTAF + phytobiotic additive (at 0.1%); and AFPA, MTAF + phytobiotic and adsorbent additive (at 0.1%). Pigs were fed the dietary treatments for 26 days. Pens had a solid floor and were equipped with two nipple drinkers and a metal feeder with three divisions.

Representative samples of contaminated corn were taken (2 kg), and two subsamples of each sample were sent for mycotoxin analysis at Romer Laboratories (Union, MO, USA) and Veterinary Diagnostic Laboratory, North Dakota State University (Fargo, ND, USA), and Alltech Inc. (Nicholasville, KY, USA). The average mycotoxin concentration obtained on the three analyses was reported for contaminated corn (Table 7) and diets (Table 8). Corn contaminated with aflatoxin B1 (3.2 mg/kg) and fumonisin B1 (160 mg/kg) was mixed with uncontaminated corn to achieve the targeted level of mycotoxins (aflatoxin: 0.19 mg/kg and fumonisin B1: 8 mg/kg) in the diets MTAF, AFP, and AFPA. Only uncontaminated corn was used in the diet CON. All diets were formulated to be isonitrogenous and isocaloric and meet the nutrient requirements for grower pigs (28 to 55 kg) suggested by the National Research Council [42]. Body weight and feed intake were measured weekly to obtain average daily gain, average daily feed intake, and gain–feed ratio.

Vilocym or Vilocym-Z was supplemented at 0.1% to the diets replacing the equal amount of corn.

### 5.2. Sample Collection and Laboratorial Analyses

One pig with closer body weight to the average body weight of each pen was selected and bled on day 28, in experiment 1, or on day 26, in experiment 2, according to procedures described by Kim et al. [4]. Blood samples were allowed to clot for 4 h and then were centrifuged at for 15 min at 3000× g (4 °C) to obtain serum. Serum samples were divided into aliquots and stored at −80 °C. In experiment 2, an aliquot of serum vial was thawed and used to measure one of the following analytes: tumor necrosis factor-α (indicator of systemic inflammation) or immunoglobulin G (indicator of general immune response).

Analytes were assessed following the procedures described by Holanda et al. [8]. Porcine tumor necrosis factor-α was measured using a commercial kit (PTA00, R&D Systems, Inc., Minneapolis, MN, USA) with a detection range of 23 to 1500 pg/mL. Porcine immunoglobulin G (E100-104, Bethyl Laboratories Inc., Montgomery, TX, USA) was measured with a detection range of 7.8 to 500 ng/mL.

Briefly, following Holanda et al. [8], tumor necrosis factor-α was measured by pipetting 50 µL of assay diluent supplied in the kit with 50 µL of samples into wells. The plate was covered with clear adhesive strip and incubated for 2 h. Each well was washed five times with 300 µL of washing buffer; 100 µL of TNF-α conjugate supplied in the kit was added, and the plate was incubated following the same specifications. Each well was washed five times with 300 µL of washing buffer; 100 µL of substrate solution supplied in the kit was added to each well, and the plate was incubated for 30 min in the dark. After incubation, 100 µL of stop solution supplied in the kit was added and wells were read at 450 and 570 nm to obtain the reading at 570 nm subtracted from 450 nm. Following Holanda et al. [8], IgG was measured by pipetting 100 µL of the respective affinity purified antibody into each well according to the kit dilution. The plate was incubated for 1 h. Each well was washed five times with 260 µL of washing buffer supplied in the kit; 200 µL of blocking buffer supplied in the kit were added, and the plate was incubated for 30 min. Each well was washed five times with 260 µL of washing buffer; 100 µL of samples was added and incubated for 30 min. Each well was washed five times with 260 µL of washing buffer; 100 µL of diluted horseradish peroxidase supplied in the kit was added, and the plate was incubated for 1 h. Each well was washed five times with 260 µL of washing buffer; 100 µL of tetramethylbenzidine substrate was added, and the plate was incubated in the dark for 15 min. Sulfuric acid (100 µL) at 0.18 M was used as a stop solution. The plate was read at 450 nm.

### 5.3. Statistical Analysis

Data were analyzed with SAS Software (version 9.3; Cary, NC, USA). Values of *p* < 0.05 were considered significant and those of 0.05 ≤ *p* < 0.10 were considered a trend. Data were analyzed on the basis of a completely randomized block design using the mixed model (Proc MIXED) with dietary treatments as the main effect and sex and initial body weight as blocking criteria. In the case of a significant difference or trend, analyses of preplanned contrasts between dietary treatments were performed using the Contrast statement.

## Figures and Tables

**Table 1:** Growth performance observed in nursery pigs fed diets with minimal deoxynivalenol contamination (CON), with additional deoxynivalenol contamination (MTD), MTD with phytobiotics (DP), MTD with phytobiotics and adsorbent at 0.1% (DPA1), or MTD with phytobiotics and adsorbent at 0.2% (DPA2) in experiment 1.

Treatment	CON	MTD	DP	DPA1	DPA2	SEM	*p*-Value
Body weight, kg							
day 0	11.6	11.6	11.7	11.6	11.6	0.1	0.885
day 7	12.8	12.5	12.8	12.8	12.5	0.3	0.848
day 14	15.6 ^a^	13.9 ^b^	14.0 ^b^	14.5 ^b^	14.3 ^b^	0.4	0.035
day 21	19.8 ^a^	15.9 ^c^	16.6 ^bc^	17.0 ^b^	17.1 ^b^	0.5	0.001
day 28	25.2 ^a^	19.7 ^c^	20.3 ^bc^	20.1 ^bc^	20.9 ^b^	0.5	0.001
Average daily gain, g/day							
days 0 to 7	181.0	133.4	152.9	171.0	133.4	32.5	0.777
days 7 to 14	396.1 ^a^	195.7 ^b^	180.5 ^b^	236.5 ^b^	253.2 ^b^	30.3	0.001
days 14 to 21	595.0 ^a^	285.2 ^c^	373.1 ^bc^	367.7 ^bc^	400.7 ^b^	37.1	0.001
days 21 to 28	778.9 ^a^	533.6 ^b^	517.1 ^bc^	437.5 ^c^	544.9 ^b^	34.5	0.001
days 0 to 28	487.8 ^a^	286.9 ^c^	305.9 ^bc^	303.2 ^bc^	333.1 ^b^	17.5	0.001
Average daily feed intake, g/day							
days 0 to 7	424.3 ^A^	308.7 ^B^	342.9 ^B^	443.1 ^A^	432.4 ^A^	37.2	0.055
days 7 to 14	478.9	367.0	508.9	560.2	506.1	53.6	0.159
days 14 to 21	791.4	574.3	709.7	715.8	710.2	84.3	0.497
days 21 to 28	1136.1	855.5	985.8	1031.9	967.8	92.3	0.326
days 0 to 28	707.7	526.4	636.8	687.7	654.1	60.2	0.268
Gain–feed ratio							
days 0 to 7	0.439	0.513	0.469	0.441	0.331	0.102	0.782
days 7 to 14	0.897 ^a^	0.601 ^b^	0.363 ^c^	0.478 ^bc^	0.515 ^bc^	0.088	0.003
days 14 to 21	0.804	0.499	0.577	0.593	0.595	0.082	0.142
days 21 to 28	0.724	0.652	0.583	0.458	0.589	0.069	0.117
days 0 to 28	0.729 ^a^	0.583 ^b^	0.508 ^b^	0.487 ^b^	0.525 ^b^	0.052	0.019

^abc^ Means lacking common superscript letters differ (*p* < 0.05). ^AB^ Means lacking common superscript letters tend to differ (0.05 ≤ *p* < 0.10).

**Table 2:** Growth performance observed in growing pigs fed diets with minimal mycotoxin contamination (CON), with additional aflatoxin and fumonisin contamination (MTAF), MTAF with phytobiotics (AFP), or MTAF with phytobiotics and adsorbent (AFPA) in experiment 2.

Treatment	CON	MTAF	AFP	AFPA	SEM	*p*-Value
Body weight, kg						
day 0	28.6	28.3	28.3	28.8	0.2	0.231
day 7	35.2	34.6	34.6	35.1	0.2	0.219
day 14	43.3 ^a^	41.4 ^b^	41.3 ^b^	41.6 ^b^	0.4	0.003
day 21	50.0 ^a^	47.9 ^b^	47.5 ^b^	48.0 ^b^	0.5	0.005
day 26	55.5 ^a^	53.4 ^b^	52.5 ^b^	53.0 ^b^	0.5	0.002
Average daily gain, kg/day						
days 0 to 7	0.939	0.895	0.900	0.899	0.026	0.611
days 7 to 14	1.158 ^a^	0.974 ^b^	0.955 ^b^	0.942 ^b^	0.045	0.008
days 14 to 21	0.960	0.930	0.881	0.911	0.031	0.355
days 21 to 26	1.100	1.087	0.998	0.986	0.054	0.333
Overall	1.035 ^a^	0.963 ^b^	0.928 ^b^	0.931 ^b^	0.018	0.001
Average daily feed intake, kg/day						
days 0 to 7	1.760 ^A^	1.716 ^AB^	1.693 ^AB^	1.650 ^B^	0.027	0.061
days 7 to 14	2.230 ^a^	1.908 ^b^	1.922 ^b^	1.905 ^b^	0.036	0.001
days 14 to 21	2.193 ^a^	2.005 ^b^	1.989 ^b^	1.962 ^b^	0.049	0.012
days 21 to 26	2.449 ^a^	2.131 ^b^	2.130 ^b^	1.992 ^b^	0.078	0.004
Overall	2.136 ^a^	1.925 ^b^	1.919 ^b^	1.869 ^b^	0.031	0.001
Gain–feed ratio						
days 0 to 7	0.533	0.524	0.532	0.545	0.012	0.687
days 7 to 14	0.520	0.507	0.498	0.495	0.016	0.706
days 14 to 21	0.441	0.467	0.444	0.465	0.014	0.462
days 21 to 26	0.451	0.514	0.468	0.489	0.028	0.418
Overall	0.486	0.499	0.485	0.497	0.009	0.559

^abc^ Means lacking common superscript letters differ (*p* < 0.05). ^AB^ Means lacking common superscript letters tend to differ (0.05 ≤ *p* < 0.10).

**Table 3:** Blood variables observed in nursery pigs fed diets with minimal deoxynivalenol contamination (CON), with additional deoxynivalenol contamination (MTD), MTD with phytobiotics (DP), MTD with phytobiotics and adsorbent at 0.1% (DPA1), or MTD with phytobiotics and adsorbent at 0.2% (DPA2) in experiment 1.

Treatment	CON	MTD	DP	DPA1	DPA2	SEM	*p*-Value
Total protein, g/dL	5.73 ^A^	5.30 ^AB^	5.18 ^B^	5.39 ^AB^	5.16 ^B^	0.15	0.090
Albumin, g/dL	2.90	2.79	2.76	2.58	2.81	0.12	0.402
Globulin, g/dL	2.83	2.51	2.41	2.81	2.35	0.19	0.277
Albumin/globulin	1.09	1.14	1.19	0.96	1.20	0.10	0.442
Aspartate aminotransferase, U/L	29.3	29.9	36.8	67.4	40.6	12.8	0.229
Alanine aminotransferase, U/L	23.8 ^C^	29.9 ^AB^	24.6 ^BC^	27.1 ^ABC^	32.0 ^A^	2.3	0.090
Alkaline phosphatase, U/L	243	195	205	192	200	17	0.232
Creatine phosphokinase, U/L	873	1161	1742	3107	1209	816	0.336
Bilirubin, mg/dL	0.10	0.10	0.10	0.11	0.10	0.01	0.422
Urea N, mg/dL	12.6 ^A^	15.1 ^B^	16.1 ^B^	16.3 ^B^	16.6 ^B^	1.1	0.070
Creatinine, mg/dL	0.70	0.71	0.74	0.78	0.74	0.04	0.761
Urea N/creatinine	18.3 ^A^	21.5 ^B^	22.4 ^B^	21.1 ^AB^	23.3 ^B^	1.2	0.074
Glucose, mg/dL	96.50	82.75	81.63	82.13	82.38	5.74	0.313
Cholesterol, mg/dL	67.9	79.6	79.8	76.8	78.1	4.8	0.392
Minerals							
P, mg/dL	11.3 ^a^	8.0 ^c^	9.7 ^b^	9.7 ^b^	9.6 ^b^	0.4	0.001
Ca, mg/dL	11.2	11.6	11.0	10.7	10.9	0.3	0.214
Cl, mEq/L	104	106	106	107	106	1	0.305
Na, mEq/L	148	146	148	147	148	1	0.455
K, mEq/L	6.41	5.70	5.83	5.71	5.75	0.21	0.113
Na/K	23.5	25.8	25.5	25.9	25.6	0.8	0.236

^ABC^ Means lacking common superscript letters tend to differ (0.05 ≤ *p* < 0.10). ^abc^ Means lacking common superscript letters differ (*p* < 0.05).

**Table 4:** Blood variables observed in growing pigs fed diets with minimal mycotoxin contamination (CON), with additional aflatoxin and fumonisin contamination (MTAF), MTAF with phytobiotics (AFP), or MTAF with phytobiotics and adsorbent (AFPA) in experiment 2.

Treatment	CON	MTAF	AFP	AFPA	SEM	*p*-Value
Total protein, g/dL	5.90	6.08	6.26	6.20	0.12	0.173
Albumin, g/dL	3.86	4.04	4.14	4.11	0.09	0.129
Globulin, g/dL	2.04	2.04	2.13	2.08	0.08	0.835
Albumin/globulin	1.93	2.01	1.98	2.01	0.09	0.896
Aspartate aminotransferase, U/L	28.0 ^b^	30.4 ^b^	33.0 ^ab^	38.4 ^a^	2.3	0.036
Alanine aminotransferase, U/L	20.9 ^b^	23.5 ^ab^	25.0 ^a^	25.4 ^a^	1.1	0.034
Alkaline phosphatase, U/L	196	207	218	223	10	0.298
Creatine phosphokinase, U/L	798	961	1029	1331	210	0.340
Bilirubin, mg/dL	0.10	0.10	0.10	0.10	0	-
Urea N, mg/dL	14.5	12.5	12.3	12.9	0.8	0.207
Creatinine, mg/dL	0.94 ^b^	1.09 ^a^	1.14 ^a^	1.12 ^a^	0.04	0.002
Urea nitrogen/creatinine	16.0 ^a^	11.6 ^b^	10.6 ^b^	11.8 ^b^	1.0	0.005
Glucose, mg/dL	91.8	97.8	91.3	94.2	3.6	0.573
Cholesterol, mg/dL	85.6 ^a^	75.8 ^bc^	78.8 ^ab^	70.4 ^c^	2.4	0.002
Immune variables						
TNF-α, pg/mL	69.1 ^b^	62.8 ^b^	80.5 ^ab^	89.8 ^a^	6.8	0.042
IgG, mg/mL	107.0	89.6	85.4	81.3	10.6	0.355
Minerals						
P, mg/dL	9.56	9.81	10.49	10.05	0.36	0.340
Ca, mg/dL	11.8	11.6	11.4	11.8	0.2	0.453
Cl, mEq/L	104	102	103	103	1	0.939
Na, mEq/L	146	147	147	147	1	0.663
K, mEq/L	5.65	5.75	5.68	5.76	0.17	0.970
Na/K	26.0	25.4	26.0	25.8	0.8	0.920

^abc^ Means lacking common superscript letters differ (*p* < 0.05).

**Table 5:** Concentration of detected mycotoxins in contaminated corn distiller’s dried grain with solubles.

Mycotoxin	Concentration, mg/kg
Deoxynivalenol ^1^	4.9
Fumonisin B1	0.8

Average of analyzed values by Veterinary Diagnostic Laboratory, North Dakota State University (Fargo, ND, USA), Romer Lab (Union, MO, USA). ^1^ Deoxynivalenol concentration is reported as sum of deoxynivalenol, 3-acetyl-deoxynivalenol, and 15-acetyl-deoxynivalenol.

**Table 6:** Feed ingredients and calculated and analyzed composition of experimental diets fed to nursery pigs with minimal deoxynivalenol contamination (CON), with additional deoxynivalenol contamination (MTD), MTD with phytobiotics (DP), MTD with phytobiotics and adsorbent at 0.1% (DPA1), or MTD with phytobiotics and adsorbent at 0.2% (DPA2) in experiment 1.

Item.	CON	MTD	DP	DPA1	DPA2
Ingredient, %					
Corn, ground yellow dent	35.4	35.4	35.4	35.4	35.4
Soybean meal, dehulled	17.0	17.0	17.0	17.0	17.0
DDGS, clean	45.0	0	0	0	0
DDGS, contaminated	0	45.0	45.0	45.0	45.0
l-Lys HCl	0.4	0.4	0.4	0.4	0.4
Salt	0.3	0.3	0.3	0.3	0.3
Vitamin–mineral mix	0.2	0.2	0.2	0.2	0.2
Limestone, ground	1.45	1.45	1.45	1.45	1.45
Monocalcium phosphate	0.05	0.05	0.05	0.05	0.05
Calculated composition:					
Dry matter, %	90.7	90.7	90.7	90.7	90.7
Metabolizable energy, kcal/kg	3324	3324	3324	3324	3324
Crude protein, %	23.8	23.8	23.8	23.8	23.8
SID Lys, %	1.01	1.01	1.01	1.01	1.01
Deoxynivalenol, mg/kg	0	2.5	2.5	2.5	2.5
Analyzed composition:					
Dry matter, %	91.4	91.2	90.8	91.0	90.6
Crude protein, %	23.1	22.9	23.2	23.4	22.8
Deoxynivalenol, mg/kg	0.3	2.6	2.7	2.8	2.7

**Table 7:** Concentration of detected mycotoxins in contaminated corn.

Mycotoxin	Concentration, mg/kg
Aflatoxin B1	3.2
Aflatoxin B2	0.47
Fumonisin B1	160

Average of analyzed values by Veterinary Diagnostic Laboratory, North Dakota State University (Fargo, ND, USA), Romer Lab (Union, MO, USA), and Alltech Inc. (Nicholasville, KY, USA).

**Table 8:** Feed ingredients and calculated and analyzed composition of experimental diets fed to growing pigs with minimal mycotoxin contamination (CON), with additional aflatoxin and fumonisin contamination (MTAF), MTAF with phytobiotics (AFP), or MTAF with phytobiotics and adsorbent (AFPA) in experiment 2.

Item	CON	MTAF	AFP	AFPA
Ingredient, %				
Corn, uncontaminated	72.5	63.5	63.5	63.5
Corn, contaminated	0.0	9.0	9.0	9.0
Soybean meal, dehulled	24.0	24.0	24.0	24.0
l-Lys HCl	0.15	0.15	0.15	0.15
l-Thr	0.05	0.05	0.05	0.05
Salt	0.30	0.30	0.30	0.30
Vitamin-mineral mix	0.2	0.2	0.2	0.2
Poultry fat	1.0	1.0	1.0	1.0
Monocalcium phosphate	0.7	0.7	0.7	0.7
Limestone	1.1	1.1	1.1	1.1
Calculated composition				
Dry matter, %	89.6	89.6	89.6	89.6
Metabolizable energy, kcal/kg	3379	3379	3379	3379
Crude protein, %	17.6	17.6	17.6	17.6
SID Lys, %	0.86	0.86	0.86	0.86
Aflatoxin, mg/kg	0	0.19	0.19	0.19
Fumonisin B1, mg/kg	0	8	8	8
Analyzed composition				
Dry matter, %	91.4	91.6	91.5	91.4
Crude protein, %	15.9	16.6	16.7	16.1
Aflatoxin B1, mg/kg	0	0.263	0.260	0.274
